# Spaceflight induces oxidative damage to blood-brain barrier integrity in a mouse model

**DOI:** 10.1096/fj.202001754R

**Published:** 2020-09-26

**Authors:** Xiao W. Mao, Nina C. Nishiyama, Stephanie D. Byrum, Seta Stanbouly, Tamako Jones, Jacob Holley, Vijayalakshmi Sridharan, Marjan Boerma, Alan J. Tackett, Jeffrey S. Willey, Michael J. Pecaut, Michael D. Delp

**Affiliations:** 1 Department of Basic Sciences, Division of Biomedical Engineering Sciences (BMES), Loma Linda University School of Medicine and Medical Center, Loma Linda, CA, USA; 2 Department of Biochemistry and Molecular Biology, University of Arkansas for Medical Sciences, Little Rock, AR, USA; 3 Arkansas Children's Research Institute, Little Rock, AR, USA; 4 Division of Radiation Health, Department of Pharmaceutical Sciences, University of Arkansas for Medical Sciences, Little Rock, AR, USA; 5 Department of Radiation Oncology, Wake Forest School of Medicine, Winston-Salem, NC, USA; 6 Department of Nutrition, Food and Exercise Sciences, Florida State University, Tallahassee, FL, USA

**Keywords:** brain tissue, blood-brain barrier, microgravity, neurodegeneration, proteomics, spaceflight

## Abstract

Many factors contribute to the health risks encountered by astronauts on missions outside Earth’s atmosphere. Spaceflight-induced potential adverse neurovascular damage and late neurodegeneration are a chief concern. The goal of the present study was to characterize the effects of spaceflight on oxidative damage in the mouse brain and its impact on blood-brain barrier (BBB) integrity. Ten-week-old male C57BL/6 mice were launched to the International Space Station (ISS) for 35 days as part of Space-X 12 mission. Ground control (GC) mice were maintained on Earth in flight hardware cages. Within 38 ± 4 hours after returning from the ISS, mice were euthanized and brain tissues were collected for analysis. Quantitative assessment of brain tissue demonstrated that spaceflight caused an up to 2.2-fold increase in apoptosis in the hippocampus compared to the control group. Immunohistochemical analysis of the mouse brain revealed an increased expression of aquaporin4 (AQP4) in the flight hippocampus compared to the controls. There was also a significant increase in the expression of platelet endothelial cell adhesion molecule-1 (PECAM-1) and a decrease in the expression of the BBB-related tight junction protein, Zonula occludens-1 (ZO-1). These results indicate a disturbance of BBB integrity. Quantitative proteomic analysis showed significant alterations in pathways responsible for neurovascular integrity, mitochondrial function, neuronal structure, protein/organelle transport, and metabolism in the brain after spaceflight. Changes in pathways associated with adhesion and molecular remodeling were also documented. These data indicate that long-term spaceflight may have pathological and functional consequences associated with neurovascular damage and late neurodegeneration.

## 1 | INTRODUCTION

The spaceflight environment, characterized mainly by ionizing radiation, microgravity, and physiological/psychological stressors, presents a significant hazard to spaceflight crews during the course of mission activities. The health risk from spaceflight-induced neuronal damage and potential adverse neurovascular effects are a major concern. To the best of our knowledge, little is known about how environmental stressors impact vascular function and blood-brain barrier (BBB) integrity. Of the various central nervous system (CNS) barriers, the BBB exerts the greatest control over the immediate microenvironment of cells in the brain. The BBB is present in all brain regions except at circumventricular organs.^1^ At these sites, blood-borne mediators can traverse the vessel wall through reduced integrity of the tight junctions (TJs) to regulate certain autonomic and neuroendocrine functions. In BBB dysfunction, typically immunological privileged sites are subjected to neuroinflammation, disruptions in the ionic microenvironment and fluid volumes, and dysregulation of neurotransmitters and neuroactive agents.^2–4^ Brain vascular endothelial cells and astrocytes mediate many of these interactions and play a critical role in regulating the BBB.^5^

Preliminary ground-based studies have shown that simulated microgravity and ionizing radiation-induced chronic endothelial dysfunction and BBB disturbance, leading to maladaptive tissue remodeling.^6,7^ Increasingly, evidence suggests that both actual microgravity, as encountered by astronauts in space, and simulated microgravity on Earth have been shown to induce many deleterious physiological effects including changes in brain structure and function.^8^ Exposure to microgravity leads to a headward shift in body fluids and alterations in cerebral fluid pressures and perfusion.^9,10^ Indeed, long-duration spaceflight has been shown to alter the brain structure and fluid distribution within the brain of astronauts.^11^ Consequently, changes in cell-cell and cell-vascular interactions could constitute a pathological mechanism(s) responsible for brain injury and neurodegeneration.^12,13^

The goal of the present study was to characterize the effects of spaceflight on oxidative stress and BBB integrity in the mouse cortex and hippocampus using immunohistochemistry (IHC), and to identify the spaceflight-induced changes in protein expression profiles in mouse brain using proteomic analysis. We hypothesized that spaceflight would induce endothelial/vascular damage, remodeling of TJ proteins, and adhesion molecules from self-perpetuating oxidative stress in the brain. These changes may contribute to BBB disturbance, ultimately, resulting in neurological deficit and neurodegeneration.

## 2 | MATERIALS AND METHODS

### 2.1 | Flight and control conditions

SpaceX-12 was launched in 2017 at the Kennedy Space Center (KSC) on a 35-day mission. Ten-week-old male C57BL/6 mice (n = 20) (Jackson laboratories, Inc Bar Harbor, ME) at the time of launch, were flown for NASA’s ninth Rodent Research experiment (RR-9) and lived in NASA’s Rodent Habitats (RH) aboard the International Space Station (ISS). All flight (FLT) mice were maintained at an ambient temperature of 26–28°C with a 12-hour light/dark cycle during the flight. Habitat ground control (GC) mice (n = 20) were kept under similar housing conditions, including temperature, humidity, and carbon dioxide (CO_2_) levels using 48-hour delayed telemetry data from the FLT group. Water and food bar diet specifically designed by NASA were provided ad libitum to FLT and GC groups. All mice received the same access to food and water. There were no significant changes in body weight before and after the mission for FLT and GC groups.^14^ NASA-Ames Research Center and KSC Institutional Animal Care and Use Committees approved this flight study. The study has been done in strict accordance with the recommendations in the Guide for the Care and Use of Laboratory Animals of the National Institute of Health (NIH).

### 2.2 | Dissection and preservation of mouse brain after spaceflight

Within 38 ± 4 hours of splashdown, the FLT mice were rapidly euthanized in 100% CO_2._ The GC mice were euthanized with the same method after 38 days of GC housing. Shortly after euthanasia, brains were removed and bisected along the midline. The right hemi-brains were placed individually in sterile cryovials, snap frozen in liquid nitrogen, and kept at -80°C prior to use. The left hemi-brains were fixed in 4% paraformaldehyde in phosphate buffered saline (PBS) for 24 hours and then, rinsed with PBS, infiltrated overnight with 30% sucrose in PBS at 4°C, and then, embedded in optimal cutting temperature (OCT) compound and frozen at -80°C for IHC and apoptosis assays. Brains were then sectioned coronally at a thickness of 20 μm unless otherwise indicated. Five systematically selected sections from each brain sample were used for each biomarker staining.^6^

### 2.3 | IHC for 4-hydroxynonenal (4-HNE)

Sections were incubated with anti-4-HNE antibody (Alpha Diagnostic International Inc, San Antonio, TX, USA) at 48°C for 2 hours followed by a fluorescence-conjugated donkey anti-rabbit IgG (catalog no. A21206, Invitrogen Corp. Waltham, MA, USA) for 2 hours at room temperature and counterstained with 4',6-diamidino-2-phenylindole (DAPI) (Invitrogen, Eugene, Oregon, USA). Images were examined using a BZ-X700 All-in-One inverted fluorescence microscope with structural illumination (Keyence Corp., Itasca, IL, USA) at 20× magnification spanning the entire cortical or hippocampal regions. Quantification of the staining is described in Section 2.7.

### 2.4 | Apoptotic cell assay

Brain sections were subjected to a terminal deoxynucleotidyl transferase dUTP nick end labeling (TUNEL) staining to characterize apoptosis. Stained sections were evaluated using the DeadEnd Fluorometric TUNEL system kit (Promega Corp., Madison, WI, USA). The same sections were then incubated with DyLight 594 Lycopersicon esculentum-Lectin (Vector Laboratories, Burlingame, CA, USA) at a 1:100 dilution for 30 minutes at room temperature to stain the endothelium. Nuclei were counterstained with DAPI (Invitrogen). Images were examined using a BZ-X700 All-in-One inverted fluorescence microscope (Keyence Corp.) at 20× magnification spanning the entire section. Quantification of the staining is described in section 2.7.

### 2.5 | IHC for aquaporin4 (AQP4) and vascular double-labeling

Sections were incubated overnight (18–21 hours) at 4°C with polyclonal rabbit anti-AQP4 (1:500, Santa Cruz Biotechnology, Inc Dallas, TX, USA) and monoclonal mouse anti-glial fibrillary acidic protein (GFAP) clone GA5 (1:1000, Millipore, Burlington, MA, USA) in 0.25% BSA, 0.25% Triton X-100 in PBS (antibody dilution buffer). Sections were washed three times in PBS and further treated with Alexa Fluor 488 goat anti-rabbit IgG and Alexa Fluor 568 goat anti-mouse IgG (1:1000, Invitrogen). Cell nuclei were counterstained with DAPI (Invitrogen) and sections were mounted and coverslipped with Vectashield Hard-Set Mounting Medium (Vector Laboratories, Burlingame, CA, USA). Images were captured with a BZ-X700 inverted fluorescence microscope (Keyence Corp.) at 20× magnification spanning the entire section. Quantification of the staining is described in Section 2.7.

### 2.6 | IHC assays for platelet endothelial cell adhesion molecule-1 (PECAM-1) and Zonula occludens-1 (ZO-1)

Sections were blocked in 1% BSA/PBS for 1.5 hours at room temperature. The vasculature was labeled with DyLight 488 Lycopersicon Esculentum Lectin (1:100, Vector Laboratories) for 30 minutes at room temperature followed by a 10 minutes wash in PBS. Sections were then incubated overnight at 4°C with rabbit anti-ZO-1 (1:50) (Thermo Fisher Scientific, Hampton, NH, USA) or CD31/PECAM-1 (1:200,Novus Biologicals, Centennial, CO, USA) in antibody dilution buffer. After three washes in PBS, sections were incubated for 1.5 hours with Alexa Fluor 568 goat anti-rabbit IgG (1:1000 in antibody dilution buffer; Invitrogen) followed by PBS washes. The cell nuclei were counterstained with DAPI solution (Invitrogen), washed in PBS, mounted on the slides, and coverslipped with Vectashield HardSet mounting medium (Vector Laboratories). Six to 10 field images were captured with a BZ-X700 inverted fluorescence microscope (Keyence Corp.) at 20× magnification spanning the entire section.

### 2.7 | Quantification of TUNEL and IHC

The numbers of TUNEL-positive cells were counted in five brain sections from each animal using ImageJ counting plugin 1.41 software (National Institutes of Health, Bethesda, MD, USA http://rsbweb.nih.gov/ij/). Cells in the cortex and hippocampus were counted separately. The surface of each random selected cortical or hippocampal region was measured on digital microphotographs using ImageJ software. The density profiles were expressed as mean number of apoptotic cells/mm^2^ across five sections per brain as a single experimental value, and counts were averaged within the hippocampus or cortex across each group (n = 6 mice/group). Data were normalized with respect to GC controls. To determine 4-HNE, AQP4, ZO-1, and PECAM immunoreactivity, fluorescence intensity was measured on randomly selected fields in the cortex or hippocampus of five brain sections in each animal and calculated using ImageJ software. Once the red channel was separated from the green and blue channels in an image, fluorescence intensities of positive staining were measured using the integral/density feature in the ImageJ program. The mean of the fluorescence intensity profile measurements across five sections per cortex or hippocampus was used as a single experimental value. Data were then extracted and averaged within the hippocampus or cortex across the group (n = 6 mice/group).

To better characterize the morphological and proliferative changes, GFAP protein expression was quantified using the integral density (IntDen) feature in the ImageJ program that measured the sum of the values of the pixels of the GFAP positive (red fluorescence area) in the image. The mean of the density across five sections per hippocampus was used as a single experimental value. The data were then extracted and averaged across each group (n = 6).

### 2.8 | Proteomics analysis

A total of 12 frozen right caudal half hemispheres containing mid- and hindbrain from GC and FLT mice (n = 6), were shipped to the Proteomics Core at the University of Arkansas for Medical Sciences for mass spectrometric analysis. The protein lysates were prepared and analyzed in the same manner as our previous study^13^ utilizing the gel-based label free quantitative approach. Briefly, the protein lysates were separated by 4%-20% Tris-Glycine SDS-PAGE (Bio-Rad Laboratories, Hercules, CA, USA). The gel was cut into 12 gel slices (2 mm thick), gel slices were destained, reduced, alkylated, and trypsin digested. Peptide products were acidified to stop the trypsin reaction. Tryptic peptides were separated by reverse phase Jupiter Proteo resin (Phenomenex, Torrance, CA, USA) on a 200 × 0.075 mm column using a nonoAcquity UPLC system (Waters Corporation, Milford, MA, USA). Peptides were eluted over a 30 minutes gradient and ionized by electrospray (2.15 kV). MS1 peptides were detected and subjected to MS/MS analysis using higher-energy collisional dissociation (HCD) on an Orbitrap Fusion Tribrid mass spectrometer (Thermo Scientific, Waltham, MA, USA) in top-speed data-dependent mode. groups using the lmFit and eBayes functions. The volcano plot was generated using base R plot function and the heatmap was generated using the ComplexHeatmap Bioconductor package^16^ in R version 4.0.0. Proteins were considered significant with an absolute fold change > 2 and a *P*-value < .05. These proteins were then analyzed using the Ingenuity Pathway Analysis (IPA; Redwood City, CA, USA www.qiagen.com/ingenuity) for pathway analysis.

A MaxQuant (Max Planck Institute, version 1.6.0.16) database search against the UniProtKB Mus musculus protein database (2018–06 release; 81,557 entries) was used to identify and quantify proteins at the MS1 precursor intensity level. The database search parameters included selecting the MS1 reporter type, trypsin digestion with up to two missed cleavages, fixed modifications for carbamidomethyl of cysteine, variable modifications for oxidation on methionine and acetyl on N-terminus, the precursor ion tolerance of 5 ppm for the first search and 3 ppm for the main search, and label-free quantitation with iBAQ. Peptide and protein identifications were accepted using the 1.0% false discovery rate identification threshold.

### 2.9 | Statistical analysis

The results obtained from TUNEL assay and IHC were analyzed by one-way analysis of variance (ANOVA) and Tukey’s HSD (honestly significant difference) test for multiple pair-wise comparisons (Sigma Plot for Windows, version 13.0; Systat Software, Inc, Point Richmond, CA, USA). *P*-values < .05 were considered significant. Means and standard error of means (SEM) are reported. The statistical analysis of proteomics results is described above.

The MaxQuant iBAQ intensities for each sample were median normalized so the medians were equal to the sample with the maximum median (GC sample 4). Median normalized iBAQ intensities were then imported into Perseus (version 1.6.1.3, Max Planck Institute) to perform log2 transformation and impute the missing values using a normal distribution with a width of 0.3 and a downshift of two standard deviations. Principal Component Analysis (PCA) was performed on the normalized dataset and a batch effect was detected among replicates due to processing samples on two different days (data not shown). The batch effect was corrected using the R function ComBat from the SVA Bioconductor package. The limma package^15^ was used to calculate differential expression among the FLT and GC sample

## 3 | RESULTS

### 3.1 | Detection of oxidative damage with 4-HNE

**FIGURE 1:**
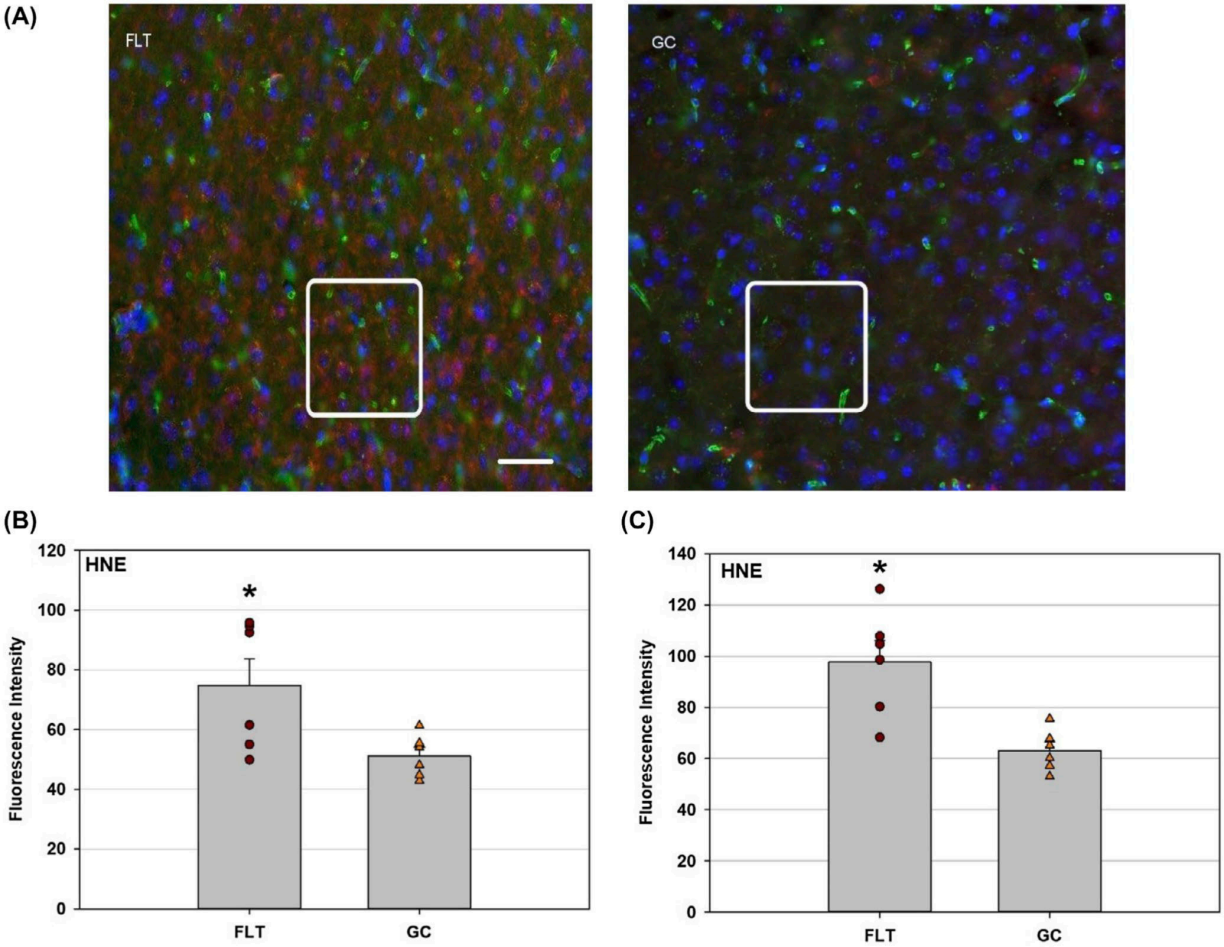
Cellular oxidative damage in the cortex and hippocampus. 4-HNE positive staining was identified with red fluorescence; the nuclei were counterstained with DAPI (blue). The vessels were stained with Lycopersicon esculentum-Lectin (green). Scale bar = 50 μm. The average fluorescence intensity of 4-HNE was measured and calculated using the ImageJ program. Values are represented as mean density ± SEM. A, Representative micrographs of brain sections in the cortex were evaluated for lipid peroxidation by immunostaining with anti-4-hydroxynonenal (4-HNE) antibody. Distinguished staining pattern was highlighted in the boxes for better comparison between FLT and GC samples. B, Increased 4-HNE staining was seen in flight (FLT) cortex compared to the ground control (GC) group (*P < .05). C, Increased 4-HNE immunoreactivity was seen in FLT hippocampus compared to the GC group (*P < .05)

The occurrence of lipid peroxidation, a marker of reactive oxygen species (ROS) formation, was evaluated using an anti-4-HNE antibody. Increased 4-HNE immunofluorescent intensity was seen in both the brain cortex (Figure 1A,B) and in the hippocampus (Figure 1C) of the FLT group, compared to the habitat GC group.

### 3.2 | Detection of apoptotic cells in the brain

**FIGURE 2:**
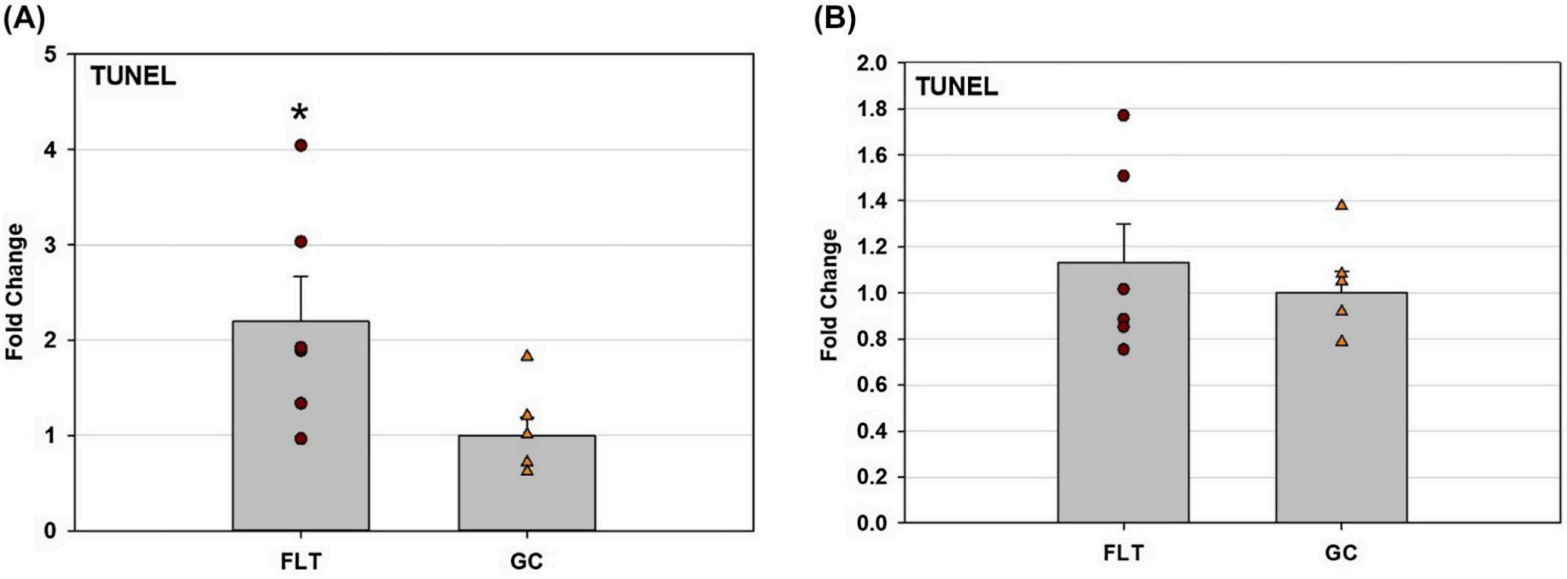
Apoptosis induced by spaceflight in the brain as measured by TUNEL assay. Data were normalized with respect to GC controls. Bar-graph showing fold increase over GC with mean ± SEM. A, Quantification of TUNEL immunoreactivity was based on density profile of TUNEL-positive cells in the brain hippocampus. *P < .05 compared to the GC group. B, Quantification of TUNEL immunoreactivity was based on density profile of TUNEL-positive cells in the brain cortex. Statistical analysis did not reveal any significant differences between groups for apoptosis in the cortex

A significant increase (average of 2.2-fold) of TUNEL positive cells were seen in the FLT hippocampus (*P* < .05) compared to the GC group (Figure 2A). The numbers of TUNEL positive cells in the cortex were statistically similar between FLT and GC groups (Figure 2B).

### 3.3 | Assessment of BBB integrity with AQP4 and GFAP

**FIGURE 3:**
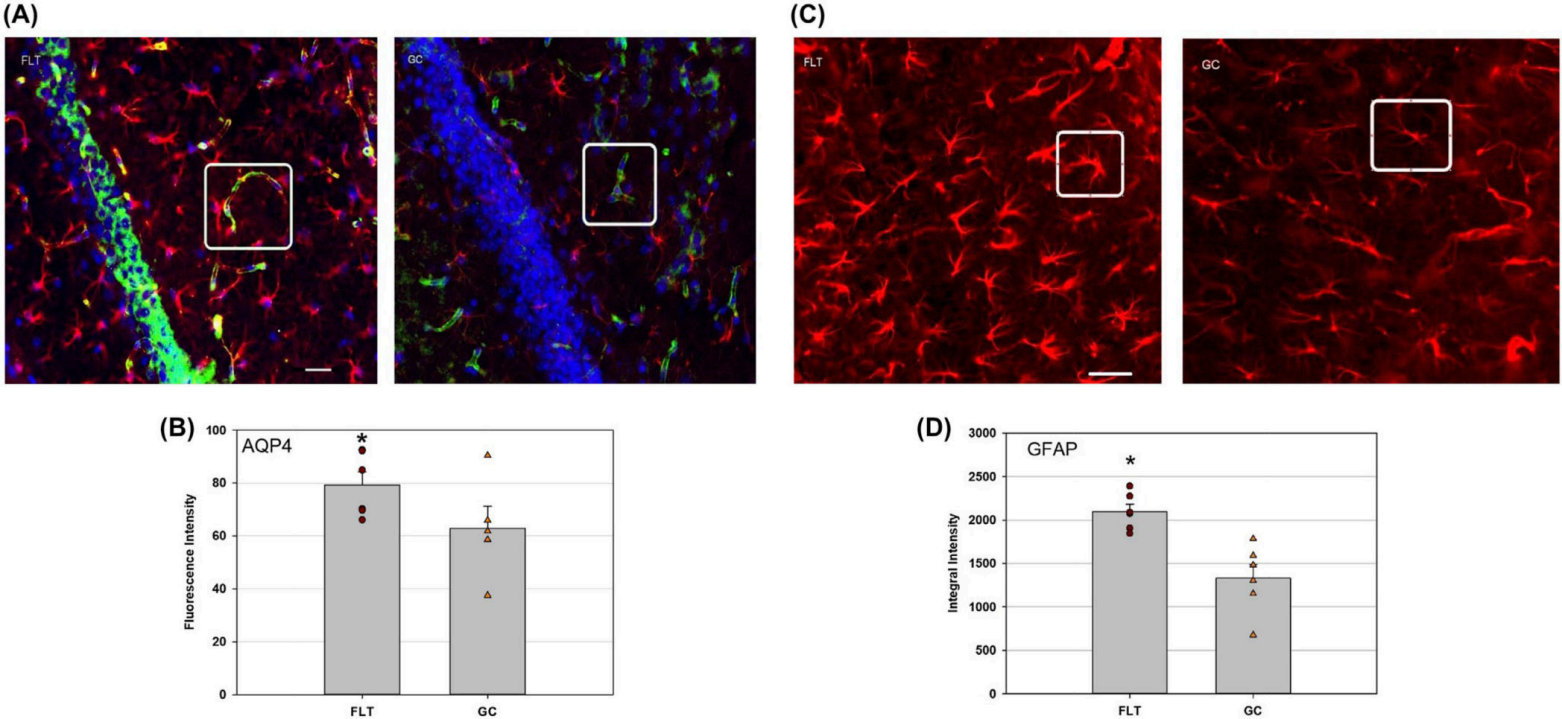
Glial fibrillary acidic protein (GFAP) and aquaporin4 (AQP4) staining in the hippocampus. A, Representative micrographs of brain sections after immunostaining with anti-GFAP and AQP4 antibodies on flight (FLT) and ground control (GC) samples. AQP4 positive staining was identified by green fluorescence, GFAP with red, and the cell nuclei with blue (DAPI). Scale bar = 50 μm. Distinguished staining pattern was highlighted in the boxes for better comparison between FLT and GC samples. B, Values are represented as mean density ± SEM. *P < .05 compared to the GC group. C, Representative images of GFAP immunoreactivity of mouse FLT hippocampus show more GFAP-positive astrocytes with larger in size than those in the GC group. Note that many GFAP-positive astrocytes exhibited hypertrophic morphology. Scale bar = 50 μm. Distinguished staining pattern was highlighted in the boxes for better comparison between FLT and GC samples. D, Quantitative analyses of the GFAP-positive immunoreactivity in the hippocampus. Values are represented as mean integrated density (IntDen, the sum of the values of the pixels in the image) ± SEM. *P < .05 compared to the GC group

Two astrocyte biomarkers, GFAP and AQP4, were used to assess the integrity and function of the BBB and microvessel damage. GFAP is present in the soma and astrocytic process and serves as a target protein for immunohistochemical detection of astrocytes. AQP4 is a water channel protein concentrated at the luminal surfaces of astrocyte end-feet, which outline the vascular bed to which they attach. In the FLT mouse hippocampus, elevations of AQP4 expression in the perivascular end-feet were demonstrated and coincided with hypertrophic perivascular GFAP-positive astrocytes (Figure 3A,B). Hippocampal GFAP-positive astrocytes after spaceflight showed a highly ramified morphology with a larger soma sizes and thickened processes compared to GC (Figure 3C). GFAP-positive astrocyte pixel density in the FLT hippocampus was significantly increased compared to GC (Figure 3D). There was no notable difference in the pattern and density of GFAP and APQ4 staining in the cortex of FLT animals compared to GC (data not shown).

### 3.4 | Evaluation of TJ protein ZO-1 and cell adhesion molecule PECAM-1

**FIGURE 4:**
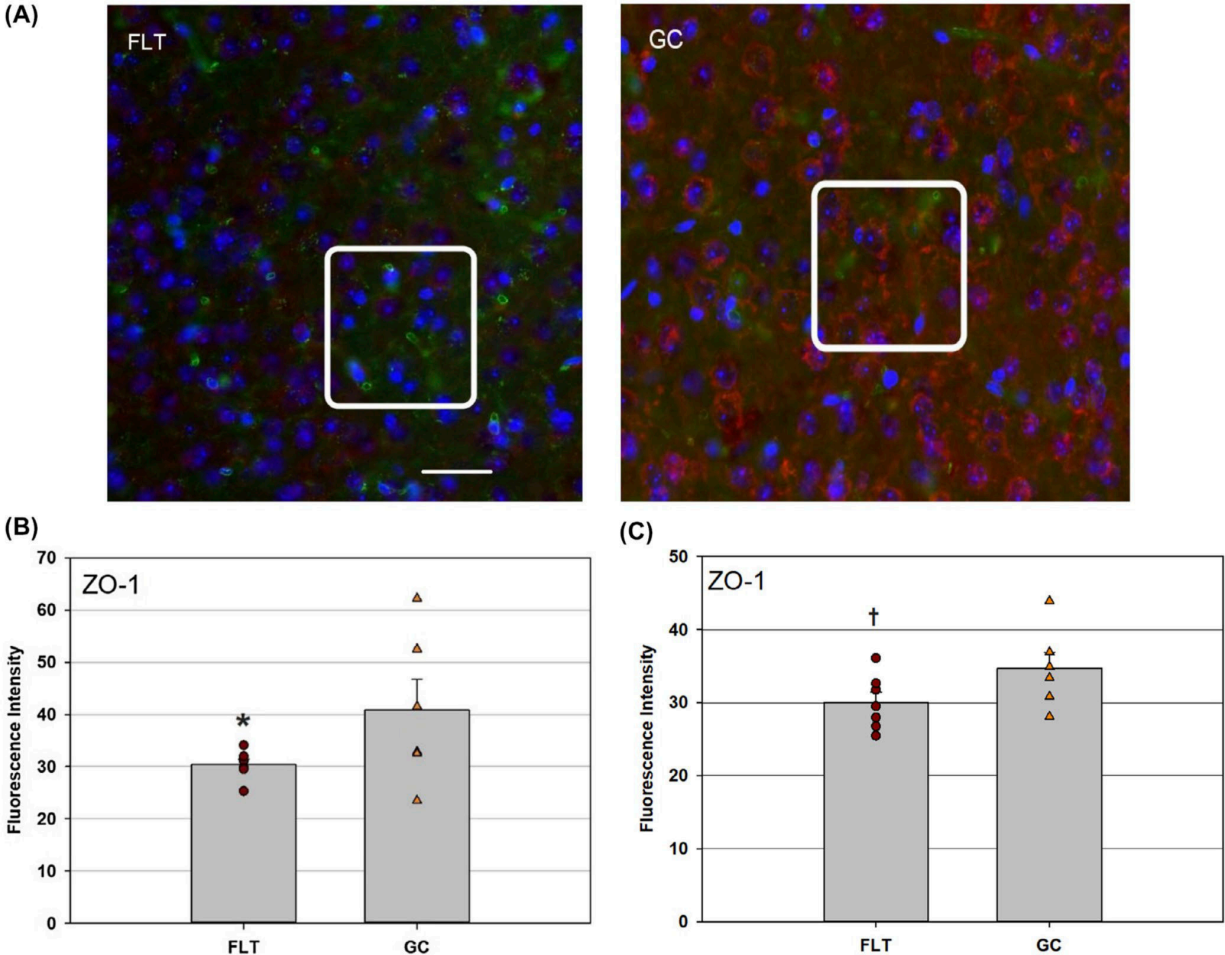
Zonula occludens-1 (ZO-1) staining in the cortex and hippocampus. A, Representative images of ZO-1 in cortical sections of flight (FLT) and ground control (GC) mice. ZO-1 positive cells were identified with red fluorescence, endothelium was stained with lectin (green). The nuclei of the cells were counterstained with DAPI (blue). In the GCcortex, positive ZO-1 staining were apparent. Only some positive cells were found in the cortical region from FLT mice. Scale bar = 50 μm. Distinguished staining pattern was highlighted in the boxes for better comparison between FLT and GC samples. B, Quantitative analyses of ZO-1 immunoreactivity in the cortex. Values are represented as mean density ± SEM. *Significantly lower than GC groups in the cortex (*P* < .05). C, Quantitative analyses of ZO-1 immunoreactivity in the hippocampus. Values are represented as mean density ± SEM. ^†^Tendency for difference between FLT and GC groups in the hippocampus (*P* = .07)

**FIGURE 5:**
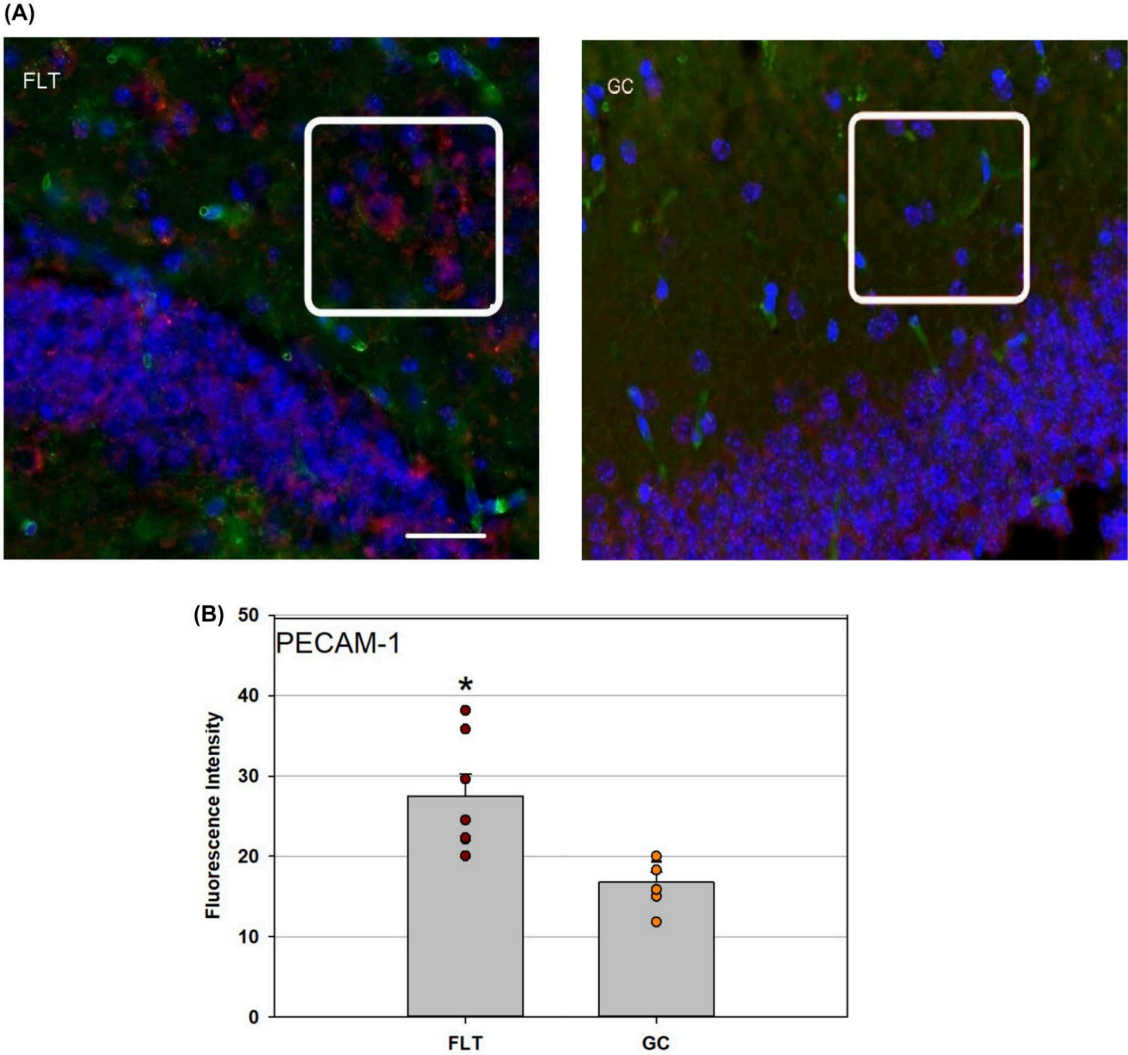
Platelet endothelial cell adhesion molecule (PECAM-1) staining in the hippocampus. A, Representative images of PECAM-1 in the hippocampus of flight (FLT) and ground control (GC) mice. PECAM-1 positive cells were identified with red fluorescence, endothelium was stained with lectin (green). The nuclei were counterstained with DAPI (blue). In the control brain tissue, only some positive cells were found in the hippocampus. In the hippocampal region of FLT mice, enhanced immunoreactivity of PECAM cells was apparent. Scale bar = 50 μm. Distinguished staining pattern was highlighted in the boxes for better comparison between FLT and GC samples. B, Quantitative analyses of PECAM-1 immunoreactivity in the hippocampus. Values are represented as mean density ± SEM. *Significantly higher than GC group in the hippocampus (*P* < .05)

Considering cell junction proteins and cell-cell adhesion molecules contribute greatly to BBB integrity, tissue expressions of ZO-1 and PECAM-1 in the brain cortex, and hippocampus were evaluated. Decreased ZO-1 immunoreactivity was detected in the FLT brain compared to the GC group (Figure 4A). The difference in ZO-1 expression between the FLT and GC groups was more pronounced in the cortex (*P* < .05, Figure 4B) than in the hippocampus (*P* = .07, Figure 4C). Furthermore, in FLT mice, increased PECAM-1 immunoreactivity was seen in the hippocampus of the FLT group compared to GC mice (Figure 5).

There were no statistical differences in PECAM-1 protein expression in the cortex between the two groups (data not shown).

### 3.5 | Proteomics analysis

**FIGURE 6:**
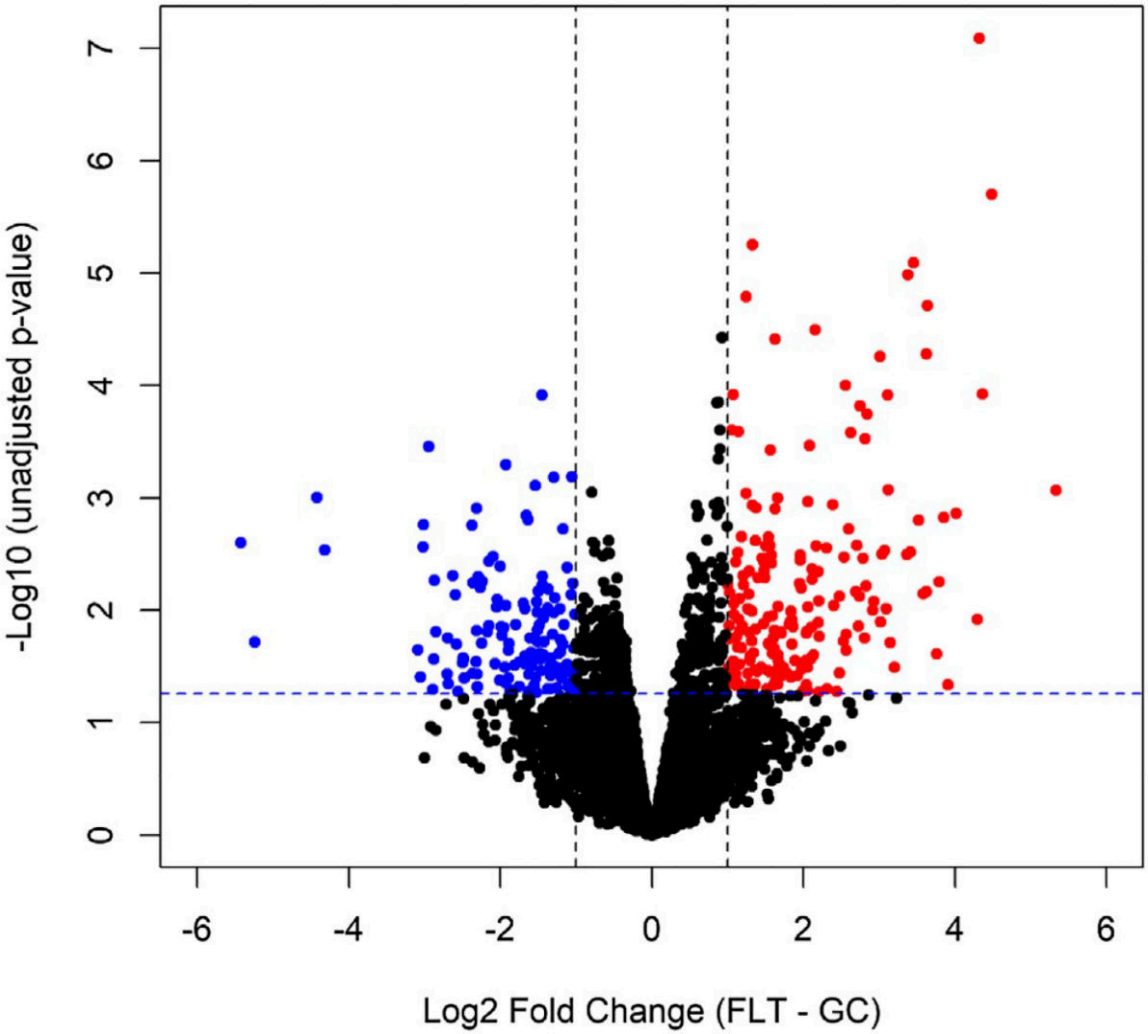
Volcano plot showing all identified proteins extracted from the right caudal half hemibrain containing mid- and hindbrain. The y-axis consists of –log_10_ unadjusted p-values based on limma, while the *x*-axis consists of the log_2_ fold change (FLT vs GC). The vertical lines indicate a fold change > 2 threshold. The horizontal line indicates a *P*-value of .05. Proteins highlighted in red were significant at a fold change > 2 and an unadjusted *P*-value < .05

**FIGURE 7:**
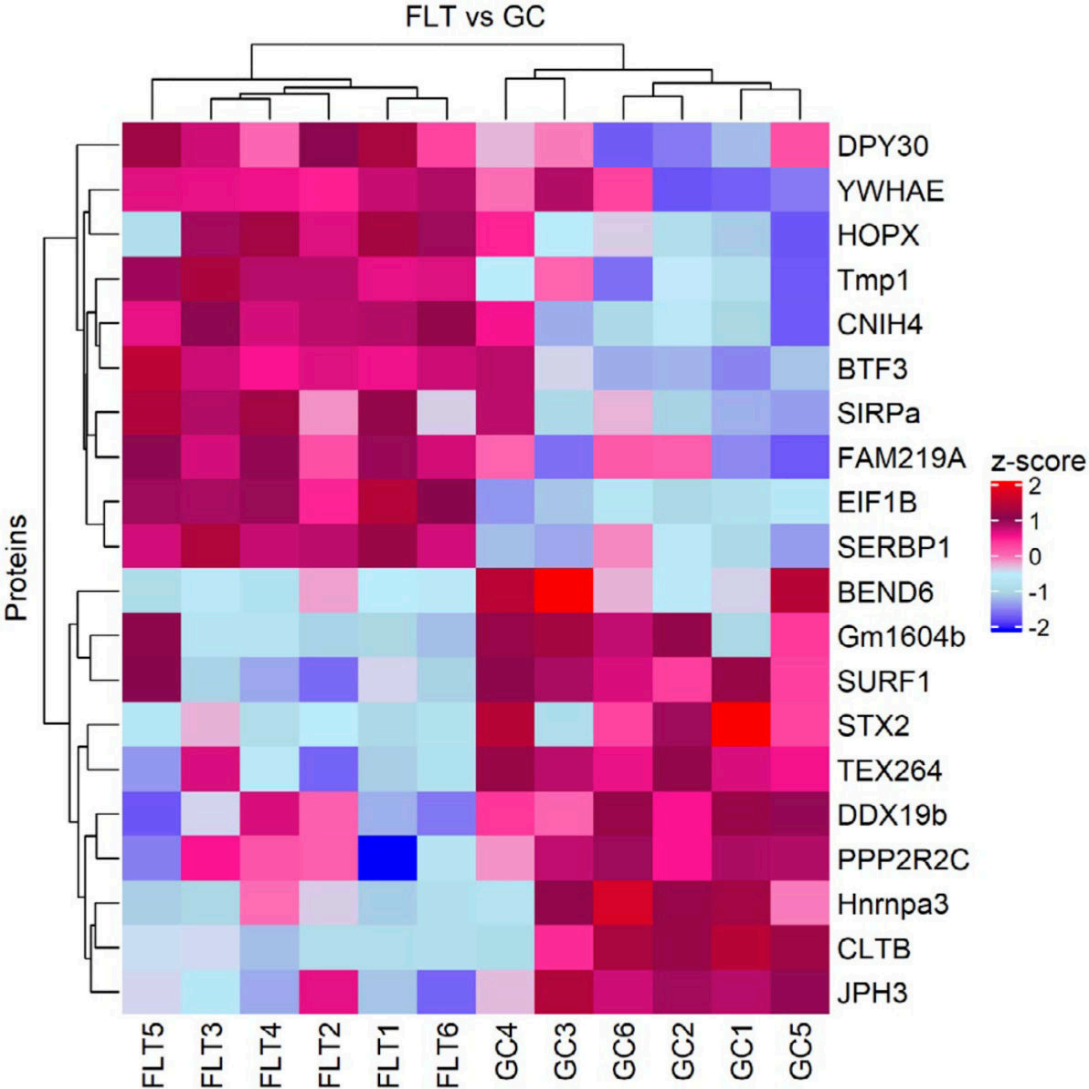
Hierarchical cluster of the top upregulated and downregulated significantly differentiated proteins between flight (FLT) and ground control (GC). The top 10 upregulated and downregulated proteins were clustered using the log2 normalized intensities scaled by z-score statistics. Proteins were considered significant with fold change > 2 and an unadjusted *P*-value < .05 and they were clustered along the top of the heatmap. Red = positive Z-score for upregulation, blue = negative Z-score for downregulation. The proteins clearly separated the two sample groups. These proteins were also shown in the volcano plot (Figure 6) above the horizontal line

A total of 6,287 proteins were identified in which 328 were significantly different comparing the FLT and GC with a *P*-value < .05 and fold change > 2 based on the Limma ebayes analysis. Volcano plots represent the overall proteins identified and their significance (Figure 6). Table 1 lists top 10 upregulated or down-regulated differentially expressed proteins by a fold change > 2 and a *P*-value < .05 identified by IPA. The hierarchical cluster heatmap was generated to visually represent the significant protein intensities of each group (Figure 7). These proteins represent the top differentially expressed proteins between FLT and GC. The heatmap shows clear separation of the two groups based on the protein expression. Quantitative proteomic analysis showed that many proteins responsible for cell function, apoptosis, mitochondrial function, nervous system development, protein/organelle transport, and metabolism were significantly altered by spaceflight.

**FIGURE 8:**
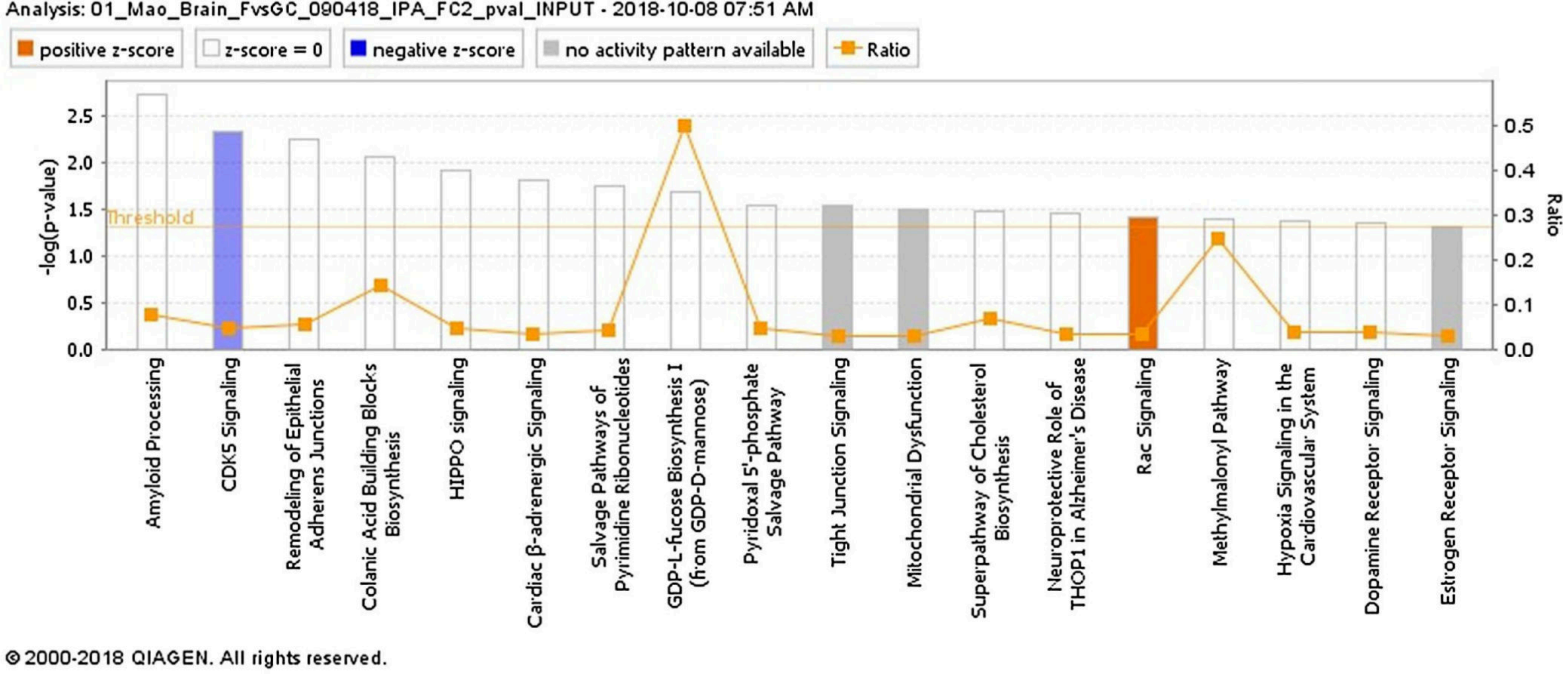
Ingenuity Pathway Analysis (IPA, https://www.qiagenbioinformaticscom/products/ingenuitypathway-analysis) of top canonical pathways altered between flight (FLT) and ground control (GC) brain tissue (-log10 (*P*-value) ≥ 1.3 = significant at *P* < .05)

To better understand the importance of identified differentially expressed proteins (DEPs), these DEPs with a fold change > 2 and a *P*-value < .05 were analyzed using IPA to identify the canonical pathways.^17^ Figure 8 summarizes the top 18 pathways that were significantly impacted by spaceflight. These pathways include adhesion molecule remodeling, tight junction signaling, and dopamine receptor signaling. Other significantly altered pathways were the neuronal apoptotic pathway involving cyclin-dependent kinase 5 (CDK5) signaling and the Hippo signaling.

Prediction of significant activation and inhibition of bio-functional activity following spaceflight was performed with IPA software based on *P* < .05 and Z-score values compared to the GC group. Z-scores were used to determine amount of functional activity with Z > 2.0 for activation or Z < -2.0 for inhibition (Table 2). Negative value of Z scores indicated negative impact of altered protein expression to the functions related to dendritic growth and branching, cell migration and coordination In accordance with our TUNEL results, key proteins with their function associated with brain cell death were significantly activated.

## 4 | DISCUSSION

Spaceflight-induced changes in measures revealing increased oxidative damage (Figure 1) and disruption in BBB integrity in the mouse brain. The data demonstrated that spaceflight-induced apoptosis in the brain (Figure 2), and evoked changes in the expression of BBB-related proteins: AQP-4 (Figure 3), GFAP (Figure 3), ZO-1 (Figure 4), and PECAM-1 (Figure 5), which all play important roles in BBB integrity and function. These protein changes suggest BBB disruption could happen during and/or following 35 days of spaceflight. We also identified spaceflight-induced changes in proteomic profiles and pathways in the brain tissues include functional changes such as cell cycle progression, apoptosis, mitochondrial function, metabolism, and behavior (Figures 6–8 and Tables 1 and 2). Given the importance of the vasculature and BBB in healthy brain function, we believe that understanding the impact of the space environment on the neurovasculature is crucial in both assessing neurodegenerative risks for astronauts during long-duration missions and the development of potential countermeasures.

The results show increased 4-HNE staining indicative of oxidative damage to the brain after spaceflight compared to GC controls. The CNS is sensitive to oxidative injury due to its high oxygen consumption,^18^ content of oxidizable unsaturated lipids^19^ and low levels of antioxidant defenses.^20^ Oxidative injury has been implicated as a causative or contributory factor in a number of neurodegenerative conditions, including aging, ischemia, and traumatic damage.^21–25^ The cerebral vasculature is particularly susceptible to oxidative stress, which is critical since cerebral endothelial cells play a major role in the creation and maintenance of the BBB.^26^ Evidence has suggested strong involvement of oxidative stress in the pathophysiology of neurodegenerative diseases.^27^ Indications of persistent oxidative stress after irradiation have also been demonstrated in the brain of mice^28^ and rats.^29^

Several lines of evidence suggest that oxidative stress is an important mediator in the body’s response to spaceflight conditions, such as the change in the gravity vector.^30,31^ Studies have shown that both simulated microgravity and spaceflight are associated with increased markers of lipid peroxidation in the brain of both humans and rodents,^32,33^ and these effects were more pronounced after long-duration spaceflight.^34,35^ Fluid shifts due to the elimination of gravitational gradients in microgravity result in altered cerebral circulation followed by oxidative stress.^36^ Although, the consequences of oxidative stress in the CNS due to spaceflight are not exactly known, acute and chronic oxidative stress in ground-based scenarios have been shown responsible for the development and progression of cerebrovascular injury.^37^

Because AQP4 is highly expressed in the perivascular astrocyte end-feet surrounding the BBB,^38^ astrocytic interactions with vascular capillaries were investigated using AQP4 labeling. The astrocytic end-feet occupy a larger surface area of the microvasculature than the neural processes.^39^ Astrocyte end-feet wrap around capillary endothelial cells and regulate brain water transport through the expression of AQP4.^1^ AQP4 is prevalent in astrocytic membranes at the blood-brain interface and plays a role in a number of pathophysiological processes.^40^ Furthermore, there are reports of cross-talk between astrocyte activation, AQP expression, and brain inflammation in response to injury.^41^ Increased AQP4 expression has been demonstrated to coincide with perivascular astrocyte swelling.^42^ Additionally, oxidative stress triggers the upregulation of AQP4 and disturbance of BBB integrity and edema formation.^43^ These observations are consistent with our findings that spaceflight concomitantly enhanced the expression of AQP4 and 4-HNE oxidative biomarkers. Similar perturbations were observed in our previous studies of simulated microgravity and low-dose radiation.^7,44^

In the present study, we also observed increased perivascular reactive GFAP astrocytes in the hippocampus of FLT mice, which indicated proliferation and hypertrophy of reactive astrocytes, a process called gliosis in response to damage to the brain.^45^ Activated astrocytes have been shown to secrete chemokines, pro-and anti-inflammatory cytokines and growth factors that are known to damage the endothelium and cause disruption of the BBB.^46^ Increases in the numbers of astrocytes with neuro-inflammation are also evident in response to oxidative stress due to fractionated radiation.^47^ However, one previous study showed that exposure to microgravity for 14 days reduced the GFAP expression in rat hippocampal astrocytes.^48^ The duration of the flight, re-adaptation period and animal species may have an impact on astrocyte response. Nevertheless, findings from the present study and others indicate that spaceflight conditions disrupt normal astrocytic homeostasis and function. However, changes in BBB integrity and barrier function need to be further studied to specifically assess BBB permeability and hemodynamics.

TJs between endothelial cells are most abundant in brain capillaries. ZO-1 is one of the most important cytoplasmic anchoring proteins of TJs.^49^ TJs are the main structures responsible for preventing the free paracellular exchange of substances between the brain parenchyma and blood.^50,51^ Decreased expression of ZO-1 in the hippocampus of FLT mice gave evidence that spaceflight may induce TJ disruption at the BBB, lead to increased permeability and contribute to brain injury. Functional and structural changes in the BBB including TJ protein expression and cell adhesion may also contribute to the onset and progression of neurodegenerative pathologies and diseases, as demonstrated in both human and mouse studies.^52–54^ Moreover, dysfunction of the BBB is a feature of aging,^55^ and increased permeability of the BBB precedes the formation of senile plaque formation in an animal model of Alzheimer disease.^56^ In addition, PECAM-1, which is a member of the immunoglobulin family of cell adhesion molecules, plays an important role in the migration of leukocytes through the endothelial layer during neuroinflammation^57^ and in the inflammatory mechanisms associated with neurodegeneration.^58^

Increased expression of PECAM-1 and subsequent BBB dysfunction may also lead to pathophysiological changes in neurological injury and disorders.^59^ It is known that neuroinflammation and disturbance of the brain barrier integrity have been implicated in the occurrence and development of several neurological diseases.^60^ Neurovascular dysfunction occurs when cell-cell junctions and cell-matrix signaling break down. Pro-inflammatory cytokines and chronic immunocyte infiltration promotes greater expression of cellular adhesion molecules and TJ disruption at the BBB.^61^ In this study, the decrease in TJ proteins together with the increase in GFAP and adhesion molecules, in conjunction with markers of apoptosis, suggests the involvement of neuroinflammation in the disruption of the BBB due to long-duration spaceflight. The likelihood of increases in the brain susceptibility to later development of neurological disorders as results of these observed changes need to be further investigated.

The Cdk5 signaling pathway was significantly downregulated in the brain upon spaceflight. Cdk5 kinase plays an important role in the CNS for regulating neuronal cell death and cognitive function. Cdk5 becomes deregulated in neurological disorders such as Alzheimer’s disease, Parkinson’s disease, and Huntington’s disease.^62^ The functional significance of Cdk5 pathway alterations due to spaceflight need to be studied in more detail.

Several oxidative stress-related signaling pathways were found to be significantly altered, including the Hippo signaling pathway, Rac signaling pathway, and dopamine signaling pathway. The Hippo signaling pathway is an important regulator of cell survival in response to oxidative stress and plays a critical role in ROS-mediated regulation of cell migration and cell junctions.^63^ Studies have also demonstrated that the Rac signaling pathway responds to enhanced vascular oxidative stress, especially due to activation of nicotinamide adenine dinucleotide phosphate (NADPH) oxidase.^64^ The dopamine receptor signaling pathway has been reported to modulate oxidative stress responses, inflammation, and apoptosis..^65^ An alteration of this signaling pathway may indicate impairment of endothelial function. These observed signaling pathway alterations are in agreement with our assessment of 4-HNE, PECAM-1, and apoptosis, which showed a significant increase in the brain after spaceflight, as well as a previous study reporting impaired endothelium-dependent vasodilation of cerebral arteries in mice after 30 days of spaceflight.^66^

Of all the proteins altered by spaceflight, signal regulatory protein alpha (SIRPα) was significantly upregulated in the FLT group by 3.6-fold compared to GC. This protein is involved in the negative regulation of adhesion of cerebellar neurons, neurite outgrowth, and glial cell attachment.^67^ It also plays a key role in intracellular signaling during synaptogenesis and in synaptic function,^68^ and acts as an important inhibitory receptor that regulates a number of leukocyte functions including regulation of neutrophil migration across epithelial monolayers and monocyte migration across the BBB.^69,70^ The upregulation of this protein observed in this study may indicate the possible development and progression of neuroinflammatory disease.

Evidence indicates that oxidative stress closely links to mitochondrial dysfunction and alterations in cellular homeostasis and damage, the result of which is known to lead to various neurological diseases and neurodegenerative disorders.^71^ Many of the mitochondrial proteins that are involved in oxidative stress response were upregulated or downregulated in the brain following spaceflight (eg, ACAD9, COA5, DNM1L, FDXR, NDUFAF5, NDUFV2, OPA1, PNPLA8, SCO1, SURF1, and TXN2). Cytochrome c oxidase assembly factor 1 (SURF1) is a protein localized to the inner mitochondrial membrane and thought to be involved in the biogenesis of the cytochrome c oxidase complex. The level of SURF1 protein expression showed a threefold decrease in FLT mice over GC. Low level expresion of SURF1- mitochondrial dysfunction in the brain may have effects on brain glucose metabolism, cerebral blood flow, and memory.^72^

Brain regional differences in stress response were also evident with spaceflight. FLT mice elicited statistically significant changes in BBB integrity biomarkers including AQP4, GFAP, and PECAM-1 in the hippocampus, but the impact was less substantial in the cortex. Previous studies have shown brain region-specific differences in response to various stressors.^6,73,74^ The hippocampus is the most vulnerable brain region under stress or other pathological conditions.^75,76^ The cellular components and structure of the cortex and hippocampus are notably different in terms of number and types of neurons, glia cell density, vascular density and topology, as well as metabolic activity. A previous study documented 13 days of exposure to a space environment caused modification of many proteins in the brain gray and white matter with minimum overlap of altered proteins between the two regions.^77^ These structural and cellular component differences may contribute to variations in tissue damage responses. In the current study, while IHC was performed in the cortex and hippocampus, the cortical region could not be separated from the hippocampus before proteomic analysis due to logistic limitations of postflight tissue processing for these RR-9 studies. Protein expression profiles in specific regions of the brain in response to spaceflight should, therefore, be a priority in future studies.

Finally, evidence in the present study of spaceflight-induced disruption of BBB integrity and proteomic alterations of signaling pathways associated with vascular endothelial dysfunction could have adverse effects beyond that of promoting possible neurodegenerative processes in the brain. For example, investigators have used whole-body mathematical modeling to examine the possible effects of impaired BBB function during spaceflight.^78^ Modeling simulations predicted that reductions in the integrity of the BBB would significantly elevate intracranial pressure,^78^ and correspondingly, elevations in intracranial pressure have been hypothesized to be related to impairments in visual acuity among astronauts returning from missions to the ISS.^10,79,80^ Therefore, if spaceflight-associated disruption of the BBB likewise occurs in humans, this could have significant adverse effects on both visual acuity and cognitive function of returning astronauts from long-duration missions.

In summary, this study demonstrated acute changes in markers of oxidative stress and BBB integrity that include altered expression of BBB properties and deceased expression of intercellular junction proteins in the mouse brain within 48 hours after return from spaceflight. Our findings may provide insight into cellular mechanisms that underlie the effects of oxidative stress-mediated structure and functional damage induced by spaceflight conditions. Such studies may point toward new therapeutic avenues for cerebrovascular dysfunction and degenerative diseases by targeting oxidative stress activity and its effects on neurovascular remodeling. In addition, changes in protein expression profiles of mitochondrial function, neuronal structure, neurovascular remodeling, behavior, protein/organelle transport, and metabolism in response to spaceflight conditions warrants further investigation. Due to animal number constraints, only male mice were used in this study. Future spaceflight investigation should be conducted to evaluate the progression and long-term effects of brain cellular and functional response following spaceflight that may lead to late neurodegeneration in both male and female animals.

## Figures and Tables

**TABLE 1:** Ten top up-and down-differentially expressed proteins in the mouse brain in response to flight (FLT) vs ground controls (GC); fold change > 2 with unadjusted *P* < .05

FLT vs GC	Protein names	Fold changes	Function
	BTF3	5.3	Posttranscriptional modification
	SERBP1	4.5	Fatty acid and lipid production
	CNIH4	4.4	Neurotransmission
	EIF1B	4.3	Cell assembly
Top upregulated	YWHAE	4.3	Signal transduction
	DPY30	4.0	Cell cycle
	FAM219A	3.9	Broad expression in brain
	HOPX	3.8	Cell proliferation
	Tmp1	3.6	Apoptosis
	SIRPα	3.6	Cell adhesion and leukocyte functions
	Gm1604b	−5.2	DNA damage and repair
	CLTB	−4.4	Cell assembly
	Hnrnpa3	−4.3	Motor neuron development
	SURF1	−3.0	Mitochondrial function
Top downregulated	JPH3	−3.0	Cell structure
	TEX264	−2.9	Transcription regulation
	STX2	−2.9	Cell assembly
	PPP2R2C	−2.9	Cell growth and division
	BEND6	−2.7	Chromatin binding
	DDX19b	−2.6	Cellular processes

**TABLE 2:** Prediction of activation or inhibition of functional activity in the brain induced by spaceflight (FLT) compared to ground control (GC) (*P* < .05, Z > 2.0 = activation or Z < −2.0 = inhibition)

Protein dataset categories	Disease or functions annotation	*P*-Value	Activation Z-score
	Outgrowth of dendrites	5.95 × 10^−5^	−2.19
Cell morphology, cellular assembly and organization	Dendritic growth/branching	2.42 × 10^−5^	−2.26
	Growth of dendrites	3.09 × 10^−4^	−2.58
Nervous system development and function	Coordination	4.37 × 10^−3^	−2.47
Cellular movement	Migration of brain cells	9.57 × 10^−6^	−2.94
Cell death and survival	Cell death of brain	2.39 × 10^−3^	2.37

## Abbreviations:

AQP4: aquaporin4
BBB: blood-brain barrier
Cdk 5: cyclin-dependent kinase 5
CNS: central nervous system
DAPI: diamidino-2-phenylindole
DEPs: differentially expressed proteins
FLT: flight
GC: ground control
GFAP: Glial fibrillary acidic protein
HCD: higher-energy collisional dissociation
IHC: immunohistochemistry
ISS: international space station
KSC: Kennedy space center
PECAM-1: platelet endothelial cell adhesion molecule
RH: Rodent Habitats
ROS: reactive oxygen species
SIRPα: signal regulatory protein alpha
SURF1: cytochrome c oxidase assembly factor
TJs: tight junctions
TUNEL: terminal deoxynucleotidyl transferase dUTP nick end labeling
ZO-1: Zonula occludens-1
4-HNE: 4-hydroxynonenal
